# Analysis of risk factors for stress cardiomyopathy after cardiac surgery

**DOI:** 10.3389/fmed.2026.1882150

**Published:** 2026-08-12

**Authors:** Huiyan Yang, Yuan Fang, Jing Wang

**Affiliations:** Department of Cardiovascular Surgery, General Hospital of Ningxia Medical University, Yinchuan, Ningxia, China

**Keywords:** cardiac surgery, cardiopulmonary bypass, logistic regression, risk factors, stress cardiomyopathy

## Abstract

**Objective:**

Stress cardiomyopathy (TTC) after open-heart surgery is rare but carries high morbidity. We aimed to identify independent perioperative risk factors.

**Methods:**

This retrospective case–control study included 25 TTC patients and 100 time-selected controls from 4,872 consecutive Cardiopulmonary bypass (CPB) cases (January 2019–December 2023). Preoperative, intraoperative, and early postoperative variables were compared. Multivariate logistic regression was used to determine independent predictors.

**Results:**

Univariate analysis revealed significant differences in age, female sex, neuropsychiatric history, mitral/tricuspid surgery, CPB time, aortic cross-clamp time, and maximum epinephrine dose (all *p* < 0.05). Postoperative cardiogenic shock was a frequent consequence (84% vs. 7%). Multivariate analysis identified four independent risk factors: female sex (OR 4.67, 95% CI 1.52–14.32), mitral/tricuspid surgery (OR 8.93, 95% CI 2.89–27.58), CPB >180 min (OR 5.14, 95% CI 1.76–15.06), and intraoperative epinephrine >0.1 μg·kg^−1^·min^−1^ (OR 6.02, 95% CI 1.95–18.57; all *p* < 0.01).

**Conclusion:**

Female sex, mitral/tricuspid surgery, prolonged CPB, and high-dose epinephrine are independent risk factors for postoperative TTC. Early echocardiographic surveillance, cautious catecholamine use, and right ventricular assessment may improve outcomes.

## 1 Introduction

Postoperative stress cardiomyopathy (Takotsubo cardiomyopathy, TTC) is increasingly recognized as a life-threatening complication after cardiac surgery (1, 2). Despite its clinical importance, data on risk factors are limited (3). We conducted this case–control study to delineate perioperative predictors of TTC in a large surgical cohort.

## 2 Materials and methods

### 2.1 Study design and patients

This single-center, retrospective case–control study was approved by the institutional review board; the need for informed consent was waived. All consecutive patients who underwent open-heart surgery with cardiopulmonary bypass (CPB) between January 2019 and December 2023 were screened (*n* = 4,872). TTC was diagnosed according to the InterTAK criteria (4): transient left ventricular regional wall motion abnormalities (apical, midventricular, or basal), a clear stress trigger, the absence of obstructive coronary disease, new ECG changes or troponin elevation, and exclusion of pheochromocytoma/myocarditis. All 25 TTC patients underwent emergency coronary angiography to rule out acute coronary syndrome; cardiac MRI was performed in two cases after stabilization. For each TTC case, four control patients (*n* = 100) who underwent surgery in the same ICU ward within ±7 days and did not develop TTC were randomly selected. No matching was performed for age, sex, or surgery type; these were adjusted in the multivariate model.

All 25 TTC cases were adjudicated by a multidisciplinary heart team comprising two cardiac surgeons, two cardiac intensivists, and a cardiologist with expertise in non-invasive imaging. The adjudication was based on a comprehensive review of clinical presentation, electrocardiographic (ECG) data, cardiac biomarkers, echocardiographic findings, invasive coronary angiography, and—where available—cardiac magnetic resonance (CMR) imaging. Symptom onset and initial presentation. The median time from intensive care unit (ICU) admission to the first clinical suspicion of TTC was 26 h (interquartile range, 12–48 h). In all 25 cases, the suspicion was raised by the occurrence of new-onset hypotension (systolic blood pressure <90 mmHg) refractory to initial fluid resuscitation and/or low-to-moderate dose vasopressor support, in the absence of obvious surgical bleeding, cardiac tamponade, or hypovolemia.

### 2.2 Data collection

The following data were extracted from electronic records: Preoperative: age, sex, BMI, hypertension, diabetes, coronary artery disease, NYHA class ≥III, LVEF, and neuropsychiatric history. Intraoperative: mitral/tricuspid surgery, CPB duration, aortic cross-clamp time, maximum epinephrine dose (μg·kg^−1^·min^−1^). Early postoperative: cardiogenic shock (SBP <90 mmHg with hypoperfusion requiring support) was recorded as an outcome, not as a predictor.the window for epinephrine dose documentation was strictly defined. The “maximum intraoperative epinephrine dose” was obtained exclusively from the anesthesia record and refers to the highest infusion rate achieved during weaning from cardiopulmonary bypass or after completion of bypass but before the patient was transferred to the ICU. Any epinephrine boluses or continuous infusions initiated in the ICU for hemodynamic fluctuations were not included in this exposure variable. The time of first clinical suspicion of TTC—defined as unexplained hypotension with new ECG changes prompting emergency bedside echocardiography that initially confirmed segmental wall motion abnormalities—occurred at a median of 26 h (interquartile range, 12–48 h) after ICU admission, whereas the maximum intraoperative epinephrine dose was administered a median of 45 min (range, 30–90 min) before ICU admission. Thus, in all cases, epinephrine exposure clearly preceded the earliest clinical and imaging evidence of TTC. To further rule out potential carry-over effects of epinephrine administered during the peri-procedural transition period, we performed a sensitivity analysis that excluded three patients who received an epinephrine bolus within two hours of ICU admission. Re-fitting the multivariate logistic regression model demonstrated that the independent association between a maximum intraoperative epinephrine dose >0.1 μg·kg^−1^·min^−1^ and postoperative TTC remained robust (adjusted OR = 5.78, 95% CI: 1.72–19.46, *p* = 0.005).

### 2.3 Statistical analysis: SPSS 26.0 was used

Normality was tested with the Shapiro–Wilk test. Continuous data are presented as the mean ± SD or median (Interquartile Range, IQR) and are compared using the t-test or the Mann–Whitney U test. Categorical data were compared with χ^2^ or Fisher’s exact test; effect sizes with 95% CIs are reported. Variables with *p* < 0.10 in univariate analysis were entered into multivariate logistic regression.

Multicollinearity was assessed (all VIF < 2). Missing data (<3%) were handled by multiple imputation. The Hosmer-Lemeshow test evaluated model fit. A two-tailed *p* < 0.05 was considered significant.

## 3 Results

### 3.1 Univariate analysis

Table 1 shows perioperative characteristics. The groups differed significantly in age, female sex, neuropsychiatric history, mitral/tricuspid surgery, CPB time, CPB >180 min, aortic cross-clamp time, and maximum epinephrine dose (all *p* < 0.05). Notably, postoperative cardiogenic shock occurred in 84% of TTC patients versus 7% of controls (*p* < 0.001), but this was treated as a clinical outcome rather than a predictor.

**Table 1:** Perioperative characteristics of the TTC and control groups.

Variable	Control (*n* = 100)	TTC (*n* = 25)	Effect size (95% CI)	*p*
Age (years)	58.2 ± 10.4	65.8 ± 9.7	MD 7.6 (3.1–12.1)	0.001
Female, *n* (%)	32 (32.0)	19 (76.0)	OR 6.58 (2.38–18.2)	<0.001
BMI (kg/m^2^)	24.6 ± 3.2	24.9 ± 3.5	MD 0.3 (−1.2–1.8)	0.675
Hypertension, *n* (%)	48 (48.0)	14 (56.0)	OR 1.38 (0.56–3.41)	0.47
Diabetes, *n* (%)	25 (25.0)	8 (32.0)	OR 1.40 (0.52–3.77)	0.473
CAD, *n* (%)	30 (30.0)	5 (20.0)	OR 0.58 (0.19–1.78)	0.316
Neuropsychiatric history, *n* (%)	11 (11.0)	9 (36.0)	OR 4.66 (1.59–13.7)	0.002
Preoperative LVEF (%)	56.1 ± 8.2	54.8 ± 9.5	MD -1.3 (−5.2–2.6)	0.495
Mitral/tricuspid surgery, *n* (%)	22 (22.0)	18 (72.0)	OR 9.07 (3.19–25.8)	<0.001
CPB time (min)	108.5 ± 32.6	196.7 ± 58.3	MD 88.2 (63.6–112.8)	<0.001
CPB >180 min, *n* (%)	12 (12.0)	17 (68.0)	OR 16.6 (5.59–49.3)	<0.001
Aortic cross-clamp (min)	75.4 ± 25.1	105.6 ± 42.3	MD 30.2 (12.5–47.9)	<0.001
Epinephrine >0.1 μg·kg^−1^·min^−1^, *n* (%)	8 (8.0)	14 (56.0)	OR 14.9 (4.76–46.9)	<0.001
Postop. cardiogenic shock, *n* (%)	7 (7.0)	21 (84.0)	OR 71.8 (18.6–278)	< 0.001

MD: mean difference; OR: odds ratio; CI: confidence interval; CAD: coronary artery disease; LVEF: left ventricular ejection fraction. Postoperative cardiogenic shock is presented as an outcome, not a predictor.

### 3.2 Multivariate logistic regression

Age, female sex, neuropsychiatric history, mitral/tricuspid surgery, CPB >180 min, and epinephrine > 0.1 μg·kg^−1^·min^−1^ were entered into the model. Female sex, mitral/tricuspid surgery, prolonged CPB, and high-dose epinephrine emerged as independent risk factors (Table 2). The model showed good calibration (Hosmer-Lemeshow χ^2^ = 5.62, df = 8, *p* = 0.690) and 91.2% accuracy.

**Table 2:** Multivariable logistic regression for postoperative TTC.

Variable	*β*	SE	Wald χ^2^	*p*	Adjusted OR (95% CI)
Female sex	1.54	0.57	7.3	0.007	4.67 (1.52–14.32)
Mitral/tricuspid surgery	2.19	0.58	14.33	<0.001	8.93 (2.89–27.58)
CPB >180 min	1.64	0.54	9.21	0.002	5.14 (1.76–15.06)
Epinephrine >0.1 μg·kg^−1^·min^−1^	1.8	0.57	9.98	0.002	6.02 (1.95–18.57)
Constant	−4.95	0.82	36.85	<0.001	—

*β*, regression coefficient; SE, standard error; Wald χ^2^, Wald Chi-square statistic; OR, odds ratio; 95% CI, 95% confidence interval.

## 4 Discussion

Our study identifies four robust independent risk factors for cardiac surgical TTC. The 0.51% incidence is consistent with prior reports (1, 2, 5). The strong female predilection mirrors the classic Takotsubo pattern and likely reflects postmenopausal catecholamine sensitivity (6). Mitral/tricuspid surgery conferred the highest risk, likely due to abrupt afterload changes and altered subvalvular mechanics (7). It should be noted that mitral/tricuspid valve surgery carried an independent OR as high as 8.93, but this estimate may be subject to residual confounding by surgical complexity and the severity of the underlying cardiac disease. Patients undergoing these procedures often have more advanced preoperative atrial and ventricular remodeling, left atrial enlargement due to chronic volume overload, variable degrees of pulmonary hypertension, and impaired right ventricular function. These factors reduce the tolerance of the ventricles to abrupt changes in afterload, and right ventricular dysfunction is not uncommon in postoperative TTC—28% of patients in our cohort had concomitant right ventricular involvement, consistent with the published literature reporting right ventricular involvement in up to 30% of postoperative TTC cases. Moreover, mitral/tricuspid surgery is typically associated with longer cardiopulmonary bypass and aortic cross-clamp times, making it difficult to completely separate the effect of the surgical procedure itself from that of prolonged CPB. Although CPB time >180 min was included as a covariate in the multivariate regression model, the independent effect of the surgical procedure may still be partially overestimated owing to their intrinsic correlation. Future multicenter studies with larger sample sizes, or the use of causal inference methods such as instrumental variable analysis or propensity score matching, are needed to more precisely delineate the direct effect of the surgical procedure itself from the relative contributions of the accompanying surgical complexity and hemodynamic status.

Prolonged CPB and high-dose epinephrine represent modifiable intraoperative factors that amplify myocardial stunning through inflammatory and β2-adrenoceptor pathways (8). We intentionally excluded postoperative cardiogenic shock from the predictive model because it is a clinical manifestation of TTC rather than a pre-existing risk factor. Its high incidence (84%) underscores the severity of this condition. In selecting inotropes or mechanical support, right ventricular (RV) involvement must be considered. As noted in recent literature, RV dysfunction may accompany up to 30% of postoperative TTC cases (3, 5). Bedside echocardiography should therefore include a focused RV assessment to guide the choice between isolated left ventricular support (e.g., Intra-aortic balloon pumping, IABP) and biventricular support (e.g., VA-ECMO). Levosimendan, a calcium sensitizer with pulmonary vasodilatory properties, may be particularly useful when biventricular failure is present. Several limitations merit discussion. The sample size (25 events) raises concern for overfitting, though our events-per-variable ratio exceeded 5, and no multicollinearity was detected. The retrospective design and reliance on bedside echocardiography (rather than routine cardiac MRI) may have missed subtle cases. Matching was by surgery date only; we addressed confounding through multivariable adjustment. Future multicenter studies should validate these findings and develop a clinical risk score.

Patients with postoperative TTC face an underappreciated acute arrhythmic risk. Due to catecholamine-mediated remodeling of myocardial ion channels and significant QTc prolongation (maximum QTc of 523 ± 38 ms in our cohort), these patients are predisposed to torsades de pointes (TdP) and ventricular fibrillation during the acute phase. However, this arrhythmogenic substrate is transient and completely reversible; in the vast majority of patients, the long-term arrhythmic risk returns to baseline after recovery of left ventricular function. Consequently, indiscriminate implantation of a permanent implantable cardioverter-defibrillator (ICD) may lead to unnecessary device-related complications and inappropriate shocks. For high-risk patients with severe left ventricular dysfunction (LVEF ≤35%), persistently marked QTc prolongation (> 500 ms), or documented hemodynamically unstable ventricular arrhythmias, the use of a wearable cardioverter-defibrillator (WCD) may be considered as a bridging protection strategy while awaiting ventricular recovery. The WCD can provide protection against lethal arrhythmias while avoiding premature permanent ICD implantation, and it allows for re-stratification of risk after ventricular function has recovered (9). The final decision regarding the need for a long-term ICD should be deferred until 3–6 months postoperatively, once it is confirmed that the LVEF has not recovered and that the patient still meets the indications for primary or secondary prevention. Emphasizing this individualized temporary protection strategy based on the reversible pathophysiology of TTC may help optimize device therapy decisions in patients with postoperative TTC and avoid overtreatment.

Furthermore, several important variables that may influence postoperative TTC—including preoperative pulmonary artery pressure, quantitative echocardiographic indices of right ventricular function (such as TAPSE and FAC), left atrial size, quantitative severity grading of valve lesions, and preoperative rhythm status—could not be included in this study. The absence of these data limits our ability to fully evaluate the potential confounding effects in the association between mitral/tricuspid valve surgery and postoperative TTC, and also prevents us from further analyzing the interplay between right ventricular function and left ventricular apical ballooning. The clinical significance of right ventricular involvement in postoperative TTC—particularly in guiding the selection of inotropic agents and the mode of mechanical circulatory support—should be an important focus of future prospective studies.

## 5 Conclusion

Female sex, mitral/tricuspid surgery, CPB >180 min, and high-dose epinephrine independently predict postoperative TTC. For at-risk patients, early echocardiography with RV evaluation, restricted catecholamine use, and timely mechanical support may improve early recognition, risk stratification, and individualized management.

## Data Availability

The original contributions presented in the study are included in the article/supplementary material, further inquiries can be directed to the corresponding author.
